# Genome Sequencing of *Bacillus subtilis* Strain XF-1 with High Efficiency in the Suppression of *Plasmodiophora brassicae*

**DOI:** 10.1128/genomeA.00066-13

**Published:** 2013-04-04

**Authors:** Shengye Guo, Zichao Mao, Yixin Wu, Kun Hao, Pengfei He, Yueqiu He

**Affiliations:** a Faculty of Agronomy and Biotechnology, Yunnan Agricultural University, Kunming, China; b College of Plant Science and Technology, Huazhong, Agricultural University, Wuhan, China

## Abstract

The genome of the rhizobacterium *Bacillus subtilis* XF-1 is 4.06 Mb in size and harbors 3,853 coding sequences (CDS). Giant gene clusters were dedicated to the nonribosomal synthesis of antimicrobial lipopeptides and polyketides. Remarkably, XF-1 possesses a gene cluster involved in the synthesis of chitosanase that is related to the suppression of the pathogen *Plasmodiophora brassicae*.

## GENOME ANNOUNCEMENT

*Bacillus subtilis*, a Gram-positive bacterium, can produce spores resistant to heat, ultraviolet rays, electromagnetic radiation, and some chemical agents (1). It can produce a variety of antimicrobial substances, including phospholipid antibiotics (2), antimicrobial proteins (3), and volatile antimicrobial substances (4), with a great potential value in the biocontrol of plant pathogenic fungi. Now, several representatives of important *B. subtilis* strains (5, 6, 7), including the model strain 168 (8, 9), have been sequenced. Here, we report the genome sequence of the plant-associated strain XF-1, with high efficiency for controlling clubroot disease of cruciferous crops, which is found worldwide and difficult to control, and suppressing many fungal pathogens of plants.

Strain XF-1 was isolated from the rhizosphere soil of Chinese cabbage (*Brassica pekinensis*) infected by *Plasmodiophora brassicae*. Its 16S rRNA sequence has 99.0% similarity to that of *B. subtilis* (10).

The genome sequencing of *B. subtilis* XF-1 was performed with a strategy involving Solexa paired-end sequencing technology. A library containing 500-bp inserts was constructed. Sequencing was performed with the paired-end strategy of 7,275-bp reads to produce 400 Mb of filtered sequences, representing 100-fold coverage, with an Illumina Solexa Genome Analyzer (GA)IIx (Beijing Genomics Institute, Shenzhen, China), and the reads were assembled into 148 contigs and 20 scaffolds using the Short Oligonucleotides Alignment Program (SOAP)*denovo* alignment tool (http://soap.genomics.org.cn/index.html#intro2). The gaps both within and between the scaffolds were filled through the sequencing of PCR products by primer walking, with the use of an ABI 3730 capillary sequencer.

The complete genome of strain XF-1 consists of a circular 4,061,186-bp chromosome with a G+C content of 43.8%. The chromosome consists of 3,853 coding sequences (CDS), 9 rRNA operons, and 77 tRNAs. Genome annotation was performed at the NCBI Prokaryotic Genomes Automatic Annotation Pipeline (PGAAP), and the GenBank nonredundant (NR), Kyoto Encyclopedia of Genes and Genomes (KEGG) (11), and Clusters of Orthologous Groups (COG) (12) databases were employed for BLASTp identification (13).

Five gene clusters, covering 3.5% of the whole genome, were involved in the nonribosomal synthesis of lipopeptides, such as surfactin and fengycin (14), the polyketide bacillaene, and the dipeptide bacilysin. The complete gene cluster for the synthesis and modification of TasA, a type of broad-spectrum antibacterial protein, was detected in XF-1 and mirrors the completely published complete sequence of the gene *tasA* (i.e., it corresponds with the gene in *Bacillus subtilis* subsp. *subtilis* 168, accession no. NP_390342.1). The chitosanase gene (*csn*) of XF-l had an 831-bp coding sequence with 99% similarity to those of *B. subtilis* 168 (15). The *sacA* gene was detected in XF-1 and shows 97% homology to the gene cluster of *B. subtilis* 168, which might explain the character of sucrose preference in this microbe (16).

### Nucleotide sequence accession number.

The complete sequence of *B. subtilis* XF-1 has been deposited in NCBI under the accession no. CP004019.
